# Comparison of Phylogeny, Venom Composition and Neutralization by Antivenom in Diverse Species of Bothrops Complex

**DOI:** 10.1371/journal.pntd.0002442

**Published:** 2013-09-12

**Authors:** Leijiane F. Sousa, Carolina A. Nicolau, Pedro S. Peixoto, Juliana L. Bernardoni, Sâmella S. Oliveira, José Antonio Portes-Junior, Rosa Helena V. Mourão, Isa Lima-dos-Santos, Ida S. Sano-Martins, Hipócrates M. Chalkidis, Richard H. Valente, Ana M. Moura-da-Silva

**Affiliations:** 1 Laboratório de Imunopatologia, Instituto Butantan, São Paulo, Brazil; 2 Laboratório de Toxinologia, Instituto Oswaldo Cruz, Fiocruz, Rio de Janeiro, Brazil; 3 Instituto Nacional de Ciência e Tecnologia em Toxinas (INCTTox/CNPq), Brazil; 4 Instituto de Matemática e Estatística, Universidade São Paulo, São Paulo, Brazil; 5 Laboratório de Fisiopatologia, Instituto Butantan, São Paulo, Brazil; 6 Universidade Federal do Oeste do Pará, Santarém, Pará, Brazil; 7 Faculdades Integradas do Tapajós, Santarém, Pará, Brazil; Universidad de Costa Rica, Costa Rica

## Abstract

In Latin America, *Bothrops* snakes account for most snake bites in humans, and the recommended treatment is administration of multispecific *Bothrops* antivenom (SAB – *soro antibotrópico*). However, *Bothrops* snakes are very diverse with regard to their venom composition, which raises the issue of which venoms should be used as immunizing antigens for the production of pan-specific *Bothrops* antivenoms. In this study, we simultaneously compared the composition and reactivity with SAB of venoms collected from six species of snakes, distributed in pairs from three distinct phylogenetic clades: *Bothrops*, *Bothropoides* and *Rhinocerophis*. We also evaluated the neutralization of *Bothrops atrox* venom, which is the species responsible for most snake bites in the Amazon region, but not included in the immunization antigen mixture used to produce SAB. Using mass spectrometric and chromatographic approaches, we observed a lack of similarity in protein composition between the venoms from closely related snakes and a high similarity between the venoms of phylogenetically more distant snakes, suggesting little connection between taxonomic position and venom composition. P-III snake venom metalloproteinases (SVMPs) are the most antigenic toxins in the venoms of snakes from the *Bothrops* complex, whereas class P-I SVMPs, snake venom serine proteinases and phospholipases A_2_ reacted with antibodies in lower levels. Low molecular size toxins, such as disintegrins and bradykinin-potentiating peptides, were poorly antigenic. Toxins from the same protein family showed antigenic cross-reactivity among venoms from different species; SAB was efficient in neutralizing the *B. atrox* venom major toxins. Thus, we suggest that it is possible to obtain pan-specific effective antivenoms for *Bothrops* envenomations through immunization with venoms from only a few species of snakes, if these venoms contain protein classes that are representative of all species to which the antivenom is targeted.

## Author Summary

Snakebite envenomation is a serious health issue in Latin America, particularly in the Amazon, where antivenom administration may be delayed due to logistic constraints. *Bothrops* snakes are involved in most of the snakebite-related accidents in Brazil. This work reports a comparative study of the toxin composition and antigenicity of the *Bothrops* venoms used to prepare the commercial antivenom and its effectiveness against the venom from *Bothrops atrox*, a prevalent Amazon species that is not included in the pool. Our data show a lack of connection between *Bothrops* taxonomic identity and venom composition. We also show that different toxins display distinct reactivity with the tested antivenom. However, the antivenom reacted similarly with each class of toxin present in the venoms of the different snakes studied. Important evidence was the neutralization of the major toxic effects of *B. atrox* venom, not included in the mixture of antigens used to produce the antivenom. Based on the observed antigenicity of the distinct protein classes of toxins, we suggest that it is possible to obtain pan-specific and efficient *Bothrops* antivenoms via immunization with venoms from a few species of snakes that are representative of the protein composition of a large number of targeted species.

## Introduction

Envenomation by snakebites, which is incorporated by the World Health Organization (WHO) in its list of neglected tropical diseases, constitutes an important worldwide public health concern, particularly in the rural areas of tropical countries as Africa, Asia and Latin America, affecting mostly agricultural workers and children [1]. The estimated number of global envenoming events exceed 400,000, with more than 20,000 fatalities [2]. In Brazil, the incidence is above 25,000 accidents/year, and the incidence in the northern region was 52.6 accidents/100,000 inhabitants in 2008 [3]. Most of the Brazilian accidents with species notification are due to vipers of the genera *Bothrops* (83.8%), *Crotalus* (8.5%) and *Lachesis* (3.4%), with only 3.4% of accidents related to the Elapidae snakes of the genus *Micrurus* [3]. Antivenoms raised in horses are the recommended treatment in Brazil.

Based on early reports [4], it was accepted that the efficacy of a specific antivenom covers bites by those snake groups with venom represented in the pool of antigens used for horse immunization for the production of that specific antivenom. Recently, the knowledge of venom toxins has increased considerably, especially due to the characterization of detailed composition of venom proteomes based on mass spectrometry. In 2007, the concept of ‘venomics’ was introduced by Calvete et al. [5] and the method was important to describe the venom composition from a great number of snake species, as revised recently [6], [7]. Then, it was possible to characterize the families of venom toxins represented in the venoms of different species of snakes [6], [7]. The implications of venomics in the rational necessary for the development of antivenoms was further supported by the ‘antivenomics’ [8], [9], that allowed the identification of venom proteins bearing epitopes recognized by one antivenom and the toxins not covered by the immune response of the hyperimmunized animal. The importance of venomics and antivenomics was readily incorporated in antivenom development, indicating the possibility of a rational design of pan-specific antivenoms combining distinct protein families in immunization pools [10]–[12].

The venom composition of many species of *Bothrops* complex is already known by venomics [13]–[27] or indirectly by transcriptomics [28]–[32]. From these studies, it has become clear that a limited number of protein families compose the venoms of *Bothrops* snakes, with snake venom metalloproteinases (SVMPs), snake venom serine proteinases (SVSPs) and phospholipases A_2_ (PLA_2_s) being the most abundant and most frequently correlated with the clinical symptoms of envenoming. SVSPs are generally thrombin-like enzymes that are involved in the coagulation disturbances observed in most patients [33]. PLA_2_s are involved in local effects and the myotoxicity observed in bites with some species [34]. SVMPs are multifunctional enzymes involved in the local and systemic symptoms of bites, such as the induction of local hemorrhage, inflammatory reaction, activation of coagulation factors and inhibition of platelet aggregation [35]. The variability in venom composition is notable and can be correlated with phylogeny [36], [37], age [38], [39], sex [40], geographical distribution [13], [40], [41] and diet [42]–[44] of the snake. However, venom variability is mostly related to the expression level of each group of toxin rather than to the presence or absence of major families of venom proteins. Moreover, within the same protein family, variability in the toxic properties may also occur when distinct functional motifs are introduced in structurally related toxins, increasing the diversity of targets that can be affected by venom toxins [45], [46]. Thus, the relevance of variability in venom composition should also be reflected in the reactivity with antivenom and its efficacy.

This problem particularly affects *Bothrops* snakes, which are diverse in their morphological and ecological traits and are distributed in different habitats throughout Latin America [47]. Due to the great diversity of *Bothrops* snakes, the systematics and phylogenetic relationships of this group are not completely resolved, and the distinction of snakes in different genera is often suggested. Based on morphology and mtDNA sequences, a broad classification of the *Bothrops* complex by Wüster et al. recognized *Bothrops* and *Bothrocophias* as independent genera [48]; furthermore, Castoe and co-authors [49] have proposed the classification of *Bothrops*, *Bothrocophias* and also *Bothriopsis* as independent genera. More recently, the *Bothrops* genus was further divided into three independent genera by Fenwick et al. [50]: *Bothropoides*, *Rhinocerophis* and *Bothrops*, representing the groups of “jararaca/neuwiedi”, “alternatus” and “jararacussu/atrox” snakes, respectively, previously recognized by Wüster et al. [48]. This classification was further questioned by Carrasco and collaborators [47], and the maintenance of *Bothrocophias* as an independent genus and synonymizing *Bothriopsis*, *Bothropoides* and *Rhinocerophis* within the *Bothrops* genus was suggested. However, according to the emerging methodology of DNA sequencing for cladistic analyses, it is reasonable to expect that further revisions of *Bothrops* systematics will be offered in the near future.

Following the classification of Fenwick and coworkers [50], several species of *Bothropoides*, *Rhinocerophis* and *Bothrops* groups are involved in snakebite envenomings, contributing to the high number of reported incidents in Brazil [3]. Antibothropic antivenoms are used in the treatment of these patients and are produced in Brazil by horse immunization with the venoms of five species of these snakes: *Bothropoides jararaca*, *Bothropoides neuwiedi*, *Rhinocerophis alternatus*, *Bothrops moojeni* and *Bothrops jararacussu*. In spite of venomics evidences showing the venom composition of several species, there are still concerns about the efficacy of *Bothrops* antivenoms in the treatment of envenomings inflicted by species whose venom is not used for animal immunization. These objections include mostly the accidents by *Bothrops atrox*, which is the snake responsible for the majority of snake bites in the Amazon, whose venom is not included in the immunization mixture. Most of these concerns arise because, in previous studies, the venoms were independently analyzed and, also, by the lack of comparative neutralization assays in the few papers showing antivenomics data for Brazilian *Bothrops* [16], [19], [26]. Thus, the complexity of the *Bothrops* group and the relevance of these species from a public health viewpoint justify the need for a multifaceted study comparing the venoms of the most relevant species and their reactivity with antivenoms in the light of recent proteomics studies.

In this study, we used a shotgun approach that allowed a simultaneous comparison of the composition of venoms collected from six species of snakes from the *Bothrops* complex, distributed in pairs from three distinct genera [50]. Fractionated venom components were tested for reactivity with the widely-used antivenom (SAB). The efficacy of the antivenom was then assessed for the neutralization of relevant symptoms of experimental envenomings by (a) *B. jararaca*, which accounts for 50% of venom composition in the immunization pool and is prevalent in the southeastern Brazil, and (b) *B. atrox*, which is not present in the immunization pool although representing a common cause of snakebite in the Amazon. The venom analysis showed that phylogenetic classification *per se* is not directly linked to venom composition. Furthermore, the antivenoms reacted equally with the toxins from the same protein family, regardless of snake phylogeny or the presence of the venom in the immunization pool used for antivenom production, highlighting new priorities when considering the selection of venoms to be used in the production of antivenoms.

## Materials and Methods

### Venoms

The venoms of *Bothropoides jararaca*, *Bothropoides neuwiedi* (*B. n. pauloensis*, *B. n. matogrossensis*, *B. n. marmoratus*, *B. n. neuwiedi* and *B. n. diporus* subspecies), *Rhinocerophis alternatus*, *Rhinocerophis cotiara*, *Bothrops jararacussu* and *Bothrops atrox* were obtained from adult snakes of both sexes kept in captivity at the Laboratório de Herpetologia, Instituto Butantan, Brazil. The venoms from more than 10 specimens of each species were pooled, freeze-dried and stored at −20°C until use. Venoms from snakes kept under captivity represented as close as possible the same pools of venoms used for antivenom production and were used for proteomics and immunoreactivity assays. For experiments involving the neutralization of *B. atrox* venom toxic activities, we used venoms from wild *B. atrox* snakes collected at the Amazonian Floresta Nacional (FLONA) do Tapajós, Pará, Brazil, under SISBio license 32098-1, aiming to get venom samples as close as possible to the ones responsible for human accidents. Eight snakes were collected in pitfalls or by active search (five males and three females, with sizes ranging from 82 to 110 cm). The snakes were extracted in the herpetarium of Faculdades Integradas do Tapajós, Santarém, Pará, Brazil, and the venom from each snake was individually lyophilized and stored frozen until use, for which a pool was generated with equal proportions of venom from each snake. The chromatographic profile of the pool of venoms from snakes collected at Floresta Nacional do Tapajós was similar to that described below for the *B. atrox* venom pooled from snakes kept under captivity (data not shown).

### Antivenoms

The antibothropic serum (SAB) was produced at the Instituto Butantan, São Paulo, Brazil in horses immunized with a mixture of the following venoms: *B. jararaca* (50%), *B. neuwiedi* (12.5%), *R. alternatus* (12.5%), *B. moojeni* (12.5%) and *B. jararacussu* (12.5%). The final preparation consists of soluble IgG F(ab′)_2_ fragments: 1 mL neutralizes the lethality of 5 mg standard *B. jararaca* venom (according to the manufacturer). Anti-jararhagin monoclonal antibodies (MAJar-3) were produced in hybridomas previously selected and maintained in our laboratory, as previously described [51]. The MAJar-3 antibodies are IgG1 isotypes and recognize conformational epitopes located on the jararhagin disintegrin-like domain. MAJar-3 neutralizes jararhagin collagen binding and hemorrhagic activity and cross-reacts with hemorrhagins from venoms of different species of viper snakes [52].

### Venomic characterization by shotgun mass spectrometry

Fifty micrograms of each venom were subjected to trypsin digestion, as previously described [53]. The tryptic digests were desalted with in-lab-generated columns packed with Poros R2 resin (Life Technologies, USA). Each of the 12 venom digests generated (6 venoms in duplicate) were analyzed in triplicate by nanoLC-MS/MS. The separation was performed on a 75 µm×30 cm column packed with a 5-µm, 200 A Magic C-18 AQ matrix (Michrom Bioresources, USA). The eluted peptides were directly injected into an LTQ/Orbitrap XL mass spectrometer (Thermo, USA) for analysis. The MS1 spectra were acquired using the orbitrap analyzer (300 to 1,700 *m/z*) at a 60,000 resolution (for *m/z* 445.1200). For each spectrum, the 10 most intense ions were subjected to CID fragmentation, followed by MS2 acquisition on a linear trap analyzer. The tandem mass spectra were extracted by RAW Xtractor (version 1.9.9.2) [54]. All of the MS/MS samples were analyzed using ProLuCID (version 1.3.1) [55]. ProLuCID was set up to search a database (forward + reverse decoy) that was built from the protein entries contained in the NCBI non-redundant database from April 29, 2012 that satisfied the following search terms criteria: “serpentes OR snakes OR snake OR venom OR venoms OR bothrops OR bothriopsis OR bothrocophias OR rhinocerophis OR bothropoides”. The database was comprised of 87,384 entries (43,692 “forward” and 43,692 “reverse decoy”). The ProLuCID search was performed with a fragment ion mass tolerance of 600 ppm and a parent ion tolerance of 70 ppm. Cysteine carbamidomethylation was specified as a fixed modification. Scaffold version 4.0.4 (Proteome Software Inc., USA) was used to validate the MS/MS-based peptide and protein identifications. The peptide identifications were accepted if they could be established at greater than 99.0% probability by the Peptide Prophet algorithm [56], with Scaffold delta-mass correction, and the protein identifications were accepted if they could be established at greater than 99.0% probability and contained at least 2 identified peptides. The protein probabilities were assigned by the Protein Prophet algorithm [57]. The acceptable false discovery rates, at the peptide and protein levels, were less than or equal to 1%.

### Venom fractionation

The venoms were fractionated by reverse-phase high-performance liquid chromatography (HPLC) according to previously described reports [16], with some modifications. Samples of 5 mg of crude lyophilized venom were dissolved in 250 µL 0.1% trifluoroacetic acid (TFA), and the insoluble material was removed by centrifugation at 18,400×*g* for 10 min at room temperature. The proteins in the soluble material were applied to a Vydac C-18 column (4.6×250 mm, 10-µm particle size) coupled to an Agilent 1100 HPLC system. The column was eluted at 1 mL/min with a gradient of 0.1% TFA in water (solution A) and 0.1% TFA in acetonitrile (solution B) (5% B for 10 min, followed by 5–15% B over 20 min, 15–45% B over 120 min, 45–70% B over 20 min and 70–100% B over 10 min). The separations were monitored at 214 nm, and the peaks were collected manually and dried in a Speed-Vac (Savant). The fractions were resuspended in PBS, and the protein concentration was estimated by OD at 280 nm in a NanoVue plus spectrophotometer (GE Healthcare).

### Venom clustering

The venoms were classified according to their toxin composition by hierarchical clustering of observations constructed using nearest neighbor linkage method (minimum Euclidean distance between items in different clusters), considering initially each observation as an individual cluster. The degrees of similarity between observations were expressed in terms of a cluster tree (dendrogram). We performed also a Principal Component Analysis (PCA) in order to understand the key toxins responsible for the venom clustering. The principal components 1 (PC1) and 2 (PC2), which were responsible for explaining more than 70% of the total variability, were calculated using the covariance matrix. The toxin composition loadings and venom scores were expressed in terms of loading and score plots. These procedures were performed in Minitab 16 software.

The variables used for clustering and PCA were the relative concentrations of each toxin family, accessed by shotgun mass spectrometry. The mean of each protein family spectral counts was normalized by the total venom counting [1,891 (*B. atrox*); 1,727 (*B. jararacussu*); 2,719 (*B. jararaca*); 2,287 (*B. neuwiedi*); 1,252 (*R. alternatus*) and 1,767 (*R. cotiara*)], distributed within the identified protein families: SVMP-I, -II and –III (snake venom metalloproteinase - classes P-I, P-II and P-III); PLA2 (phospholipase A2); SVSP (snake venom serine proteinase); CLEC (C-type lectin); CLECL (C-type lectin-like); LAAO (L-amino acid oxidase); NGF (nerve growth factor); HYALU (hyaluronidase); VEGF (vascular endothelial growth factor); CRISP (cysteine-rich secretory protein); PDIEST (phosphodiesterase 1); ECTONT (ecto-5′-nucleotidase); PLB (phospholipase B); GLUTCYC (glutaminyl cyclase) and ACTIN (actin).

The venoms were also analyzed by the relative mAU of the highest peaks collected in C-18 reverse-phase chromatography in the elution time intervals of 56–57, 57–58, 58–60, 67–71, 108–112, 113–116, 121–123, 124–127, 128–129, 130–132, 134–136, 136–138, 139–140, 140–150, 150–152, 153–155, 157–159, 160–162, 163–164, 164–166, 166–168, 169–170, and 171–172 minutes. The mAU values of the peaks were normalized in % by the mAU of the highest peak eluted in the chromatography, taken as 100%.

### ELISA assays

Samples containing 100 µL whole venom (10 µg/mL) or isolated fractions (1 µg/mL), in carbonate buffer (pH 9.6), were used to coat maxisorb microplates (Nunc). To determine the antibody titers, plates coated with whole venom were incubated with serial dilutions of SAB (from 1∶10,000), followed by incubation with anti-horse IgG labeled with peroxidase (1∶2,000). For assessing the antigenicity of the fractions, the plates were incubated with a fixed dilution of SAB (1∶1,000) or MAJar-3 (1∶50), followed by incubation with anti-horse IgG (1∶1,000) or anti-mouse IgG (1∶1,000) labeled with peroxidase. The reactions were developed with ortho-phenylenediamine/H_2_O_2_ as the enzyme substrate, and the products were detected at 490 nm. The reactions were performed in duplicates in three independent experiments. The results of antivenom titration are expressed as mean ± sd of the six OD values. The results of fraction antigenicity were calculated as mean of the six OD values after normalization using as 100% the maximal OD value obtained in each of the independent experiments [(Fraction OD/maximal OD of the test)×100].

### Western blotting

Samples of crude venom (10 µg) were subjected to 12.5% sodium dodecyl sulfate-polyacrylamide gel electrophoresis (SDS-PAGE) under non-reducing conditions. After SDS-PAGE, the separated proteins were transferred to nitrocellulose membranes, which were then immersed in a blocking solution (5% non-fat milk in Tris-saline). The membranes were incubated with SAB (1∶1,000) as the primary antibody and then with peroxidase-labeled goat anti-horse IgG (1∶1,000). The reactive bands were detected by incubation with 4-chloro-α-naphthol and H_2_O_2_. The results shown represent three independent experiments.

### Antivenom efficacy

For accessing the neutralization of the lethal and hemorrhagic venom activities, Swiss mice bred and maintained at the Instituto Butantan (Brazil) animal house were used as an animal model.

For the neutralization of hemorrhagic activity, doses of 10 µg *B. jararaca* or *B. atrox* venom were incubated with SAB at ratios of 1, 2 or 4 times the SAB volume required to neutralize 10 µg of reference venom, according to the manufacturer. The mixtures were incubated at 37°C for 30 min, and a 50-µL aliquot of each mixture was injected intradermically in the dorsa of a group of 5 mice. The control groups included mice injected with PBS or with venom incubated with PBS. At three hours after the injection, the mice were sacrificed by CO_2_ inhalation; the skin of the dorsa was removed, and the hemorrhagic spots were measured (longest diameter multiplied by the diameter perpendicular to it). The results represent the values obtained for 5 different mice and are expressed as the % neutralization using as 100% activity the value obtained after an injection with venom incubated with PBS.

For the neutralization of lethal activity, the LD_50_ values of *B. jararaca* and *B. atrox* venoms were estimated according to previous studies [58] to avoid unnecessary animal sacrifice. In all experiments, 3 LD_50_ doses of *B. jararaca* (105 µg) or *B. atrox* (225 µg) venom were incubated with SAB at ratios of 1, 2 and 4 times the potency reference value (1 mL/5 mg venom). The mixtures were incubated at 37°C for 30 min, and 500-µL aliquots were injected intraperitoneally in groups of 5 mice. Control groups included mice injected with PBS or with venom incubated with PBS. Lethality was recorded over a period of 48 hours. The results shown represent the values obtained in 3 independent experiments and are expressed as the % neutralization considering the number of dead/live mice after 48 hours.

The neutralization of the coagulant activity was determined as previously described [59], with some modifications. Samples containing 2 minimum coagulant doses of *B. jararaca* (71.3 µg/mL) or *B. atrox* (21.7 µg/mL) venom were incubated with several dilutions of SAB for 30 min at 37°C. Each mixture was added to 100 µL bovine plasma, and the clotting times were recorded using a model ST4 mechanical coagulometer (Diagnostica Stago). Neutralization was expressed as the effective dose (ED), defined as the antivenom/venom ratio at which the clotting time was increased threefold when compared to the clotting time of plasma incubated with venom alone.

### Ethics statement

All experiments involving mice were approved by the Ethical Committee for Animal Research of the Instituto Butantan (CEUAIB), São Paulo, Brazil, (application approval number 752/10), who certified its agreement with the Ethical Principles in Animal Research adopted bt the Brazilian College of Animal Experimentation (COBEA).

## Results and Discussion

**Figure 1:**
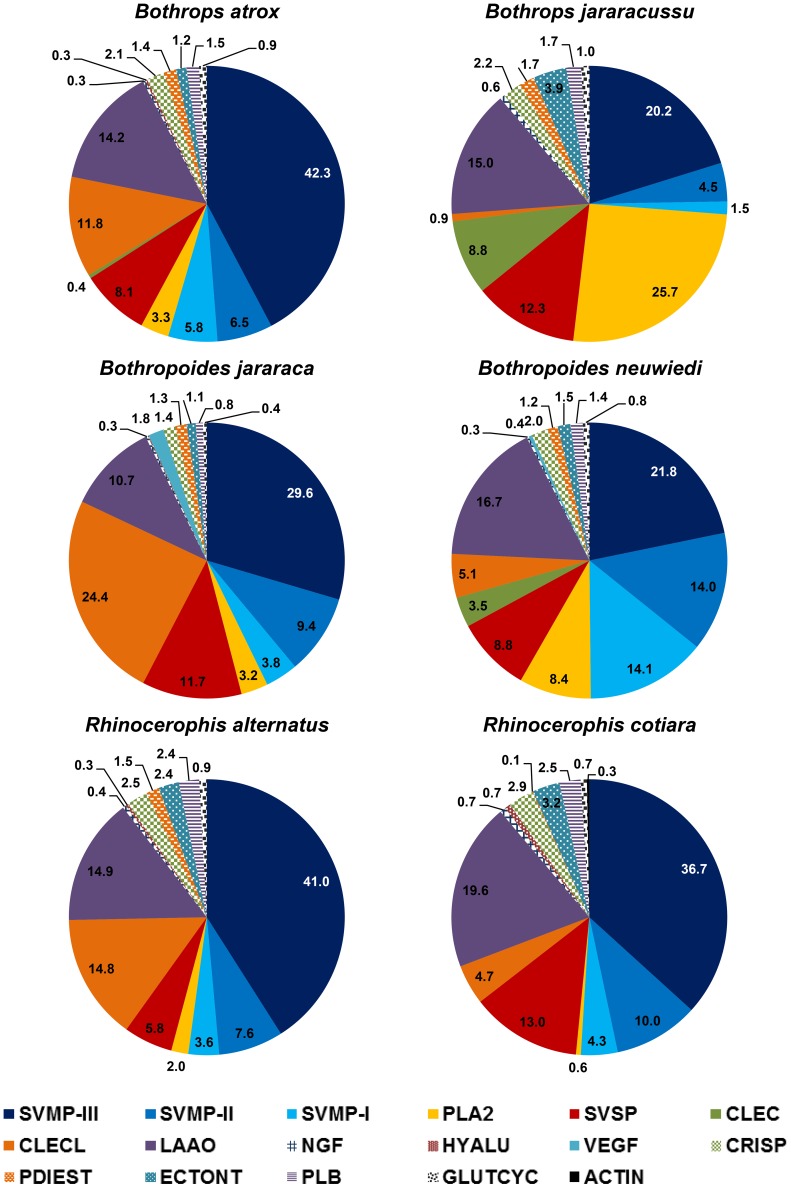
Protein family distribution for the venoms of the three different snake genera, as determined using a shotgun proteomics approach. Each venom sample was prepared in duplicate, and the MS analyses were performed in triplicate for each venom sample replicate (a total of six MS analyses per venom). The data represent the mean of the normalized total spectral count distributed as follows: 1,891 (*B. atrox*); 1,727 (*B. jararacussu*); 2,719 (*B. jararaca*); 2,287 (*B. neuwiedi*); 1,252 (*R. alternatus*) and 1,767 (*R. cotiara*). The following were identified: SVMP-I, SVMP-II and SVMP–III (snake venom metalloproteinase - classes P-I, P-II and P-III); PLA2 (phospholipase A2); SVSP (snake venom serine proteinase); CLEC (C-type lectin); CLECL (C-type lectin-like); LAAO (L-amino acid oxidase); NGF (nerve growth factor); HYALU (hyaluronidase); VEGF (vascular endothelial growth factor); CRISP (cysteine-rich secretory protein); PDIEST (phosphodiesterase 1); ECTONT (ecto-5′-nucleotidase); PLB (phospholipase B); GLUTCYC (glutaminyl cyclase) and ACTIN (actin).

To evaluate the relationship between venom composition and phylogenetic position of the species, we analyzed the proteome of the venoms from the six selected species using shotgun nanoESI-LTQ/Orbitrap. The distribution of the protein families in selected venoms was calculated according to the normalized total spectral counts. As shown in Figure 1, the data analysis revealed 15 different protein groups in different proportions: SVMP-I, -II and –III (snake venom metalloproteinase - classes P-I, P-II and P-III); PLA2 (phospholipase A2); SVSP (snake venom serine proteinase); CLEC (C-type lectin); CLECL (C-type lectin-like); LAAO (L-amino acid oxidase); NGF (nerve growth factor); HYALU (hyaluronidase); VEGF (vascular endothelial growth factor); CRISP (cysteine-rich secretory protein); PDIEST (phosphodiesterase 1); ECTONT (ecto-5′-nucleotidase); PLB (phospholipase B); GLUTCYC (glutaminyl cyclase) and ACTIN (actin). The SVMPs were the most abundant toxins in all of the venoms, particularly in the *B. atrox*, *R. alternatus*, *R. cotiara* and *B. jararaca* venoms, in which class P-III was notably the predominant toxin. PLA_2_s predominated in the *B. jararacussu* venom and was found in significant amounts in the *B. neuwiedi* venom. A significant contribution of C-type lectin-like proteins was also detected in the *B. jararaca*, *R. alternatus* and *B. atrox* venoms, whereas the SVSPs and LAAOs were almost equally distributed in all of the venoms. One interesting fact was the significant contribution of C-type (true) lectins in the *B. jararacussu* (8.8%) and *B. neuwiedi* (3.5%) venoms, in parallel with its absence (<1%) in the other venoms (Figure 1). Comparing these data with previous venomics studies [16], [18]–[20], [23], the major venom protein families as SVMPs, PLA2s and SVSPs were detected in our study in equivalent proportions. However, shotgun nanoESI-LTQ/Orbitrap allowed the detection in all venoms tested of some proteins not yet described as PDIEST, ECTONT, PLB and GLUTCYC. Also, NGF, detected here in all venoms, and HYALU, present in *B. atrox*, *B. jararaca*, *R. alternatus* and *R. cotiara* venoms, were previously detected in transcriptomes of *B. jararacussu* and *Bothropoides pauloensis*, respectively [19], [30], but not in their venomes. Five spectra identified as actin were detected in *R. cotiara* venom shotgun and due to the high sensitivity of the method, may derive from a minor contamination of the venom with venom gland cells. The most striking difference was the presence of significant amounts of LAAO, CLECL and CLEC spectra detected in our samples, compared to the previous venomics studies. Proteomics by shotgun nanoESI-LTQ/Orbitrap is based on a whole venom digestion by trypsin and the peptide mixture is then fractionated and analyzed in a high sensitive detection system. This approach may bias peptides with higher ionizable efficiency, but all protein families will be represented in the original mixture at the same proportions as they are present on venoms and the bias due to ionization efficiency will be the same for similar peptides present on venoms from different species. Thus, this method is appropriate for comparative studies, allowing the simultaneous analysis of different venoms, under exactly the same conditions. On the other hand, the traditional venomics [5] includes one step in which proteins are quantified and selected after SDS-PAGE separation, according to their staining by Coomassie blue. After trypsinization of selected bands, peptide detection and protein identification will also depend on peptide ionizable efficiency. It is well known that proteins present in venom mixtures in low proportions are hardly detectable by SDS-PAGE as some other venom proteins may be weakly stained. These proteins would be neglected in total protein detection and also when calculating their proportional participation in venom composition. The differences in protein separation methods and sensitivity of detection systems could explain the higher participation of some protein families described in our study when compared to the traditional venomics.

**Figure 2:**
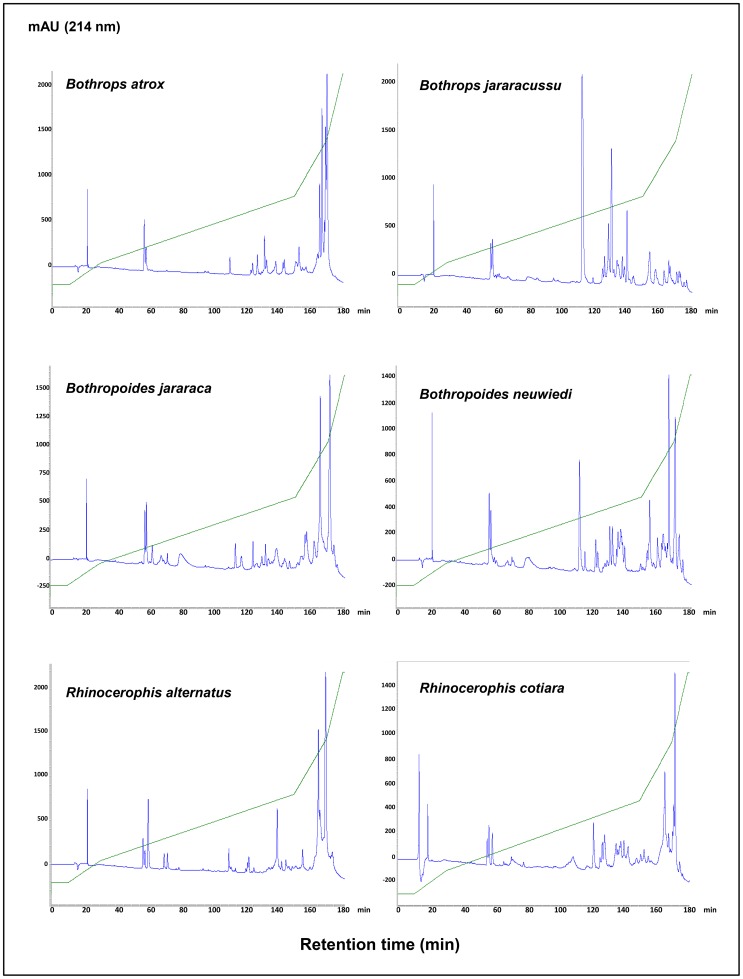
Comparison of the elution profiles of venoms from snakes classified in different genera. Samples containing 5 mg of crude lyophilized venom from *Bothrops atrox*, *Bothrops jararacussu*, *Bothropoides jararaca*, *Bothropoides neuwiedi*, *Rhinocerophis alternatus* and *Rhinocerophis cotiara*, species maintained at Instituto Butantan herpetarium, were applied to a Vydac C-18 column (4.6×250 mm, 10-µm particle size) coupled to an Agilent 1100 HPLC system. The fractions were eluted at 1 mL/min, with a gradient of 0.1% TFA in water (solution A) and 0.1% TFA in acetonitrile (solution B) (5% B for 10 min, followed by 5–15% B over 20 min, 15–45% B over 120 min, 45–70% B over 20 min and 70–100% B over 10 min). The separations were monitored at 214 nm.

The venoms were also compared according to the elution profile from reverse-phase C-18 columns. To compare our findings with the previous data from *B. atrox*, *B. cotiara* and *B. neuwiedi* venomics studies [16], [19], [20], C-18 reverse-phase chromatography protocols using similar columns, buffer systems and elution conditions were used to fractionate the venoms. Figure 2 shows the chromatographic profile of the venoms from the six species selected for this study. As expected, the venoms presented comparable chromatographic profiles to those reported in the referenced studies. According to these previous studies, the major protein families were eluted as follows: disintegrins at approximately 50–60 min [19], [20]; basic PLA_2_s at approximately 110–120 min [19]; P-I SVMPs, some D-49 PLA_2_s and SVSPs between 120 and 160 min [18]–[20] and P-III SVMPs predominating after 160 min [18]–[20]. Using these data as references, P-III SVMPs appeared to be the most abundant antigens in the chromatograms of the *B. atrox*, *R. alternatus*, *R. cotiara*, *B. jararaca* and *B. neuwiedi* venoms, whereas several different peaks in the region corresponding to P-I SVMPs and SVSPs were detected. These observations are consistent with our venomic analysis results shown in Figure 1 and with previous proteomic studies in which P-III SVMPs comprised more than 50% of *B. atrox* venom [16], [18], approximately 50% of *R. alternatus* venom [23], approximately 70% of *R. cotiara* venom [20] and approximately 25.9% of *B. neuwiedi* venom [19]. SVMPs were also reported to comprise 53.1% of *B. jararaca* venom gland toxin transcripts [31]. The *B. jararacussu* venom was the most distinct venom in this group, showing a predominant peak in the PLA_2_ region and a low abundance of SVMPs, which is consistent with the literature showing a high expression of PLA_2_ in *B. jararacussu* venom glands and representing 35% of the total transcripts, followed by only 16% SVMPs and 2% SVSPs [30]. The marked difference in *B. jararacussu* venom compared to the other *Bothrops* species was previously reported [60], and a K-49 myotoxin yield of 25% from the crude venom was purified and considered to be the predominant antigen of the *B. jararacussu* venom [61].

According to the independent parameters used to compare the venoms, in *Bothrops*, the *B. jararacussu* profile was very different from that of *B. atrox*, showing a higher content of phospholipase A_2_ and a smaller amount of the class P-III metalloproteinase (SVMP) group, as detected either by proteomics or by the elution profile of the native proteins. Within the *Bothropoides* genus, major differences were observed by proteomics, such as the higher content of CLECL and P-III SVMP in *B. jararaca* and PI and PII SVMPs, PLA_2_ and CLEC in *B. neuwiedi*. The venoms were more similar within the *Rhinocerophis* genus, particularly when comparing the elution profile of the native proteins, though a higher contribution of CLECL was found in *R. alternatus*, and higher contents of L-amino acid oxidase and serine proteinase were detected in the *R. cotiara* venom using the proteomics approach. However, the distribution of *B. atrox* venom components was very similar to that of *R. alternatus* by both methods. Furthermore, the pattern observed for *B. neuwiedi* was closer to that of *B. jararacussu* venom due to the presence of higher levels of PLA_2_ and CLEC (Figures 1 and 2). Thus, apparently, venom composition was not related to the phylogenetic position of the snakes.

**Figure 3:**
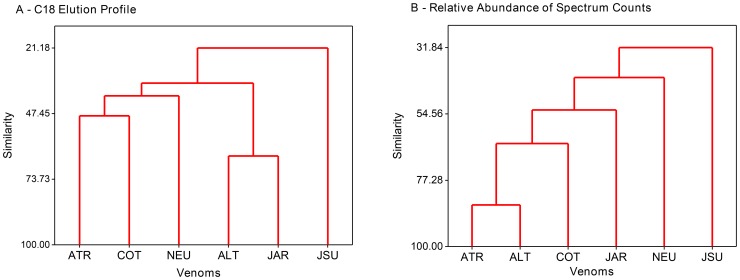
Venom clustering according to toxin composition. The venoms from *Bothrops atrox* (ATR), *Bothrops jararacussu* (JSU), *Bothropoides jararaca* (JAR), *Bothropoides neuwiedi* (NEU), *Rhinocerophis alternatus* (ALT) and *Rhinocerophis cotiara* (COT) were classified according to their protein composition by hierarchical clustering of the observations, including as a variable the normalized maximal mAU at 214 nm in defined elution intervals of C-18 reverse-phase chromatography (Panel A) or normalized total spectral counts of each protein group, as evaluated by shotgun mass spectrometry (Panel B). The procedure used an agglomerative hierarchical method linked by the minimum Euclidean distance between an item in one cluster and an item in another cluster (nearest neighbor) using the Minitab 16 software.

**Figure 4:**
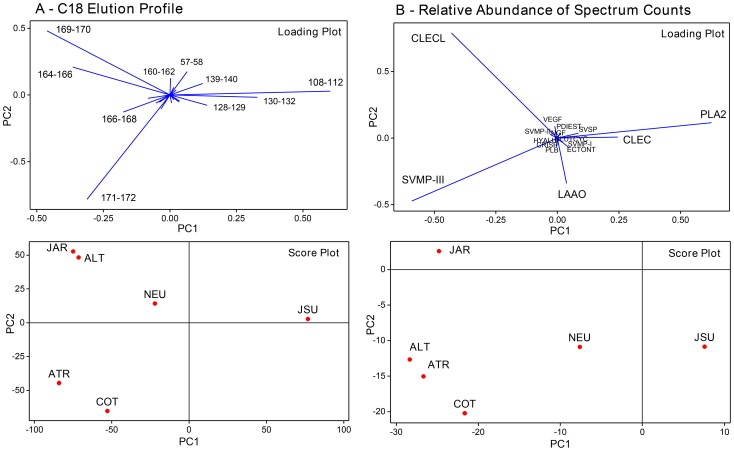
Principal Component Analysis relative to toxin composition. Loading (top) and score (bottom) plots of the principal components 1 and 2 of the venoms from *Bothrops atrox* (ATR), *Bothrops jararacussu* (JSU), *Bothropoides jararaca* (JAR), *Bothropoides neuwiedi* (NEU), *Rhinocerophis alternatus* (ALT) and *Rhinocerophis cotiara* (COT) according to their protein composition including as variables the normalized maximal mAU at 214 nm in defined elution intervals of C-18 reverse-phase chromatography (Panel A), or the normalized total spectral counts of each protein group, as evaluated by shotgun mass spectrometry (Panel B). The Principal Component Analysis was based on the covariance matrix and all calculations were carried out in the software Minitab 16.

In order to statistically demonstrate these differences, the normalized values of the venom composition obtained by the total spectrum counts of each protein family, and the mAU values of the major peaks eluted in different volumes during the C-18 chromatography, were used as variables to cluster the venoms of snake species. A Principal Component Analysis (PCA) was also carried out in order to understand the key toxins responsible for the venom clustering. The resulting dendrograms and loading and score plots of the PCA are shown in Figures 3 and 4, respectively. Clustering according to the C-18 elution profile shows a strong similarity between *R. alternatus* and *B. jararaca* venoms. *B. atrox* and *R. cotiara* venoms also show similar elution profile, but different than *R. alternatus* and *B. jararaca* venoms, forming, therefore, two different clusters. On the other hand, *B. neuwiedi* and *B. jararacussu* venoms reveal lower similarity with the two former clusters, with *B. jararacussu* having the most distinct features (Figure 3). In the PCA, shown in Figure 4A, components with most prominent loadings that contributed to venom clusterization are the fractions eluted after 160 min with the highest negative values of PC1 (Fraction 164–166: PC1 = −0.365, PC2 = 0.209; Fraction 166–168: PC1 = −0.175, PC2 = −0.128; Fraction 169–170: PC1 = −0.461, PC2 = 0.481; Fraction 171–172: PC1 = −0.311, PC2 = −0.783). These fractions were characterized mostly as class P-III SVMPs in other studies [18]–[20] and reacted with MAJar-3 monoclonal antibodies in this study (see below). Fractions with the highest PC1 positive values were eluted between 108–112 min (PC1 = 0.630, PC2 = 0.029), recognized as PLA_2_s in previous studies [19], and fractions between 130–132 min (PC1 = 0.330, PC2 = −0.018), characterized as class P-I SVMP in the venom of adult *B. atrox* from El Paují (Orinoquia, Venezuela) that underwent ontogenetic variation [16]. With respect to proteomic data, *B. atrox* and *R. alternatus* venoms were the most closely related, and distances to this group increased gradually for *R. cotiara*, *B. jararaca*, *B. neuwiedi* and *B. jararacussu* venoms. The clustering of *B. atrox* and *R. alternatus* venoms is related to high values of CLECL and P-III SVMPs, which are the proteins with most prominent loadings (CLECL: PC1 = −0.431, PC2 = 0.789, P-III SVMPs: PC1 = −0.592, PC2 = −0.472), and low values of PLA_2_ and CLEC, also with significant loadings (PLA_2_: PC1 = 0.245, CLEC : PC1 = 0.245). *R. cotiara* venom shows similar pattern with respect to P-III SVMP, PLA_2_ and CLEC, but low values of CLECL and high values of LAAO (PC1 = 0.037, PC2 = −0.339). On the other hand, *B. jararaca* venom reveals low values of LAAO and large values of CLECL. *B. neuwiedi* and *B. jararacussu* venoms present an opposite pattern, with high values of PLA_2_ and CLEC and low values of PIII-SVMP (Figure 4 B).

The dendrograms and PCAs obtained using the distinct sets of variables do not coincide, as they were based in distinct parameters. The number of total spectral counts of a given protein is not necessarily related to its mAU 214; moreover, chromatographic fractions represent mixtures of protein families treated as independent variables in the cluster corresponding to the proteomic data. In spite of these differences, both sets of variables indicate that the distribution of venoms is not related to the phylogenetic position of the snakes. It is important to note that a more comprehensive study using venoms from a larger number of species, quantitative assays for isolated components and also complete sequences of venom proteins would be essential to a definitive support of the lack of connection referred to above. Nevertheless, our data are supported by the literature. Taken together, the clusterization and PCA analysis indicate a polarization among the venoms. According to significant PC1 loadings, *B. atrox*, *R. alternatus*, *R. cotiara* and *B. jararaca* venoms are clearly opposite to *B. jararacussu* venom, the former group with prominent negative PC1 values of class P-III SVMPs, while *B. jararacussu* venom shows a polarization towards the presence of PLA_2_s and class P-I SVMPs. The same toxin polarization has been indicated to venoms from snakes that conserved the paedomorphic characteristics in their venoms (first group) and venoms of snakes whose venom underwent ontogenetic variation (in our study, *B. jararacussu* venom) [13], [16], [18], [38], [39]. Interestingly, *B. neuwiedi* venom was grouped closer to *B. jararacussu* in the cluster analysis, but showed smaller negative PC1 scores, in opposition to *B. jararacussu* venom. According to the distances, *B. neuwiedi* venom apparently conserved the paedomorphic phenotype, but may be suffering a transition to the ontogenetic changes observed in *B. jararacussu* or *B. atrox* from Colombia.

Correlations between phylogeny and venom composition have been appointed in the literature [36], [37]. Nevertheless, differences in composition of venoms from snakes belonging to the same genera are also present in the literature [62]–[64]. In a recent study, Gibbs et al. [65] found no evidence for significant phylogenetic signal in venom variation of *Sistrurus spp*, suggesting that diet variation may play a more important role in molding the venom composition. A remarkable variation in venom composition and toxicity was reported for rattlesnakes from *Crotalus viridis/oreganus* complex [66] and *Crotalus durissus* and *Crotalus simius* in Central and South American species [67]. In the latter, differences were related to the conservation of the newborn characteristics of Central American rattlesnake, *C. simus*, in the South American species and sub-species of *C. durissus*, a typical example of paedomorphism [67]. These examples are also found in snakes of the *Bothrops* complex. Tashima et al. [20] reported significant differences in venom composition between two species closely related, *R. cotiara* and *R. fonsecai.* A paedomorphic characteristic was also conserved along the dispersion of *B. atrox* from Central America to the Brazilian Amazon [16], including in the population used in this study. The conservation of the paedomorphic characteristics in *B. atrox* accounted for the concentration of class P-III SVMPs, which greatly contributes to the overall toxicity of *Bothrops* venoms [35]. Paedomorphic characteristics were not conserved in *B. jararacussu* venom, which has predominance of enzymatically inactive myotoxic PLA_2_s [60] and therefore, presents lower toxicity compared to *B. atrox* venom. The difference in composition and toxicity of *B. atrox* and *B. jararacussu* venoms argues in favor that the gain in toxicity was favorable in *B. atrox* due to its smaller size. According to this hypothesis, paedomorphic characteristic would not be essential to *B. jararacussu* snake that is very large and capable of inoculating large amount of venoms in mammalian preys.

**Figure 5:**
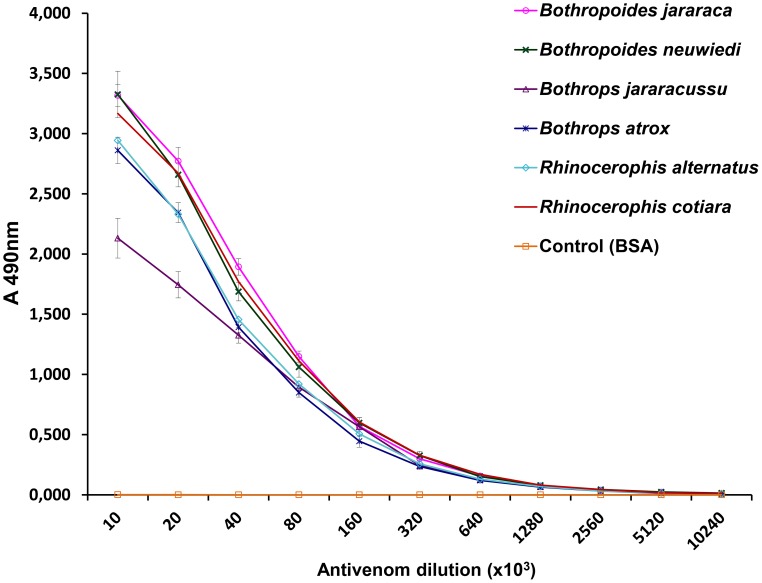
Comparison of ELISA titration curves of *Bothrops* antivenom with venom from snakes classified in different genera. Samples containing 100 µL whole venom (10 µg/mL) were used to coat maxisorb microplates (Nunc), which were incubated with crescent dilutions of SAB (starting from 1∶10,000), followed by incubation with anti-horse IgG labeled with peroxidase (1∶2,000). The reactions were developed with ortho-phenylenediamine/H_2_O_2_ as the enzyme substrate, and the products were detected at 490 nm. The experiments were performed in duplicate in three independent experiments, and the results are expressed as the mean ± sd of the six OD values.

**Figure 6:**
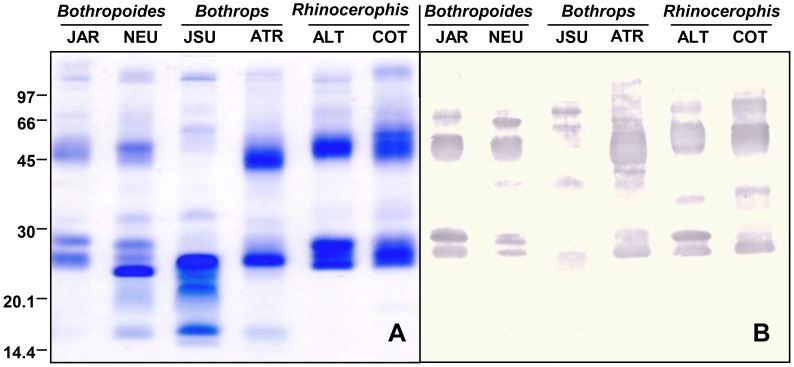
Comparison of electrophoretic profile (A) and *Bothrops* antivenom antigenic reactivity (B) of venoms from snakes classified in different genera. Samples containing 10 µg *Bothropoides jararaca* (JAR), *Bothropoides neuwiedi* (NEU), *Bothrops atrox* (ATR), *Bothrops jararacussu*(JSU), *Rhinocerophis alternatus* (ALT) and *Rhinocerophis cotiara* (COT) venoms were fractionated by SDS-PAGE (12.5% acrylamide gels) under non-reducing conditions and were either stained with Coomassie blue (**A**) or transferred to nitrocellulose membranes, which were then incubated with SAB (1∶1,000) as the primary antibody and peroxidase-labeled goat anti-horse IgG (1∶1,000). The reactive bands were detected by incubation with 4-chloro-α-naphthol and H_2_O_2_ (**B**). The numbers at the left indicate the mobility of the molecular mass markers in kDa. These results represent three independent runs.

**Figure 7:**
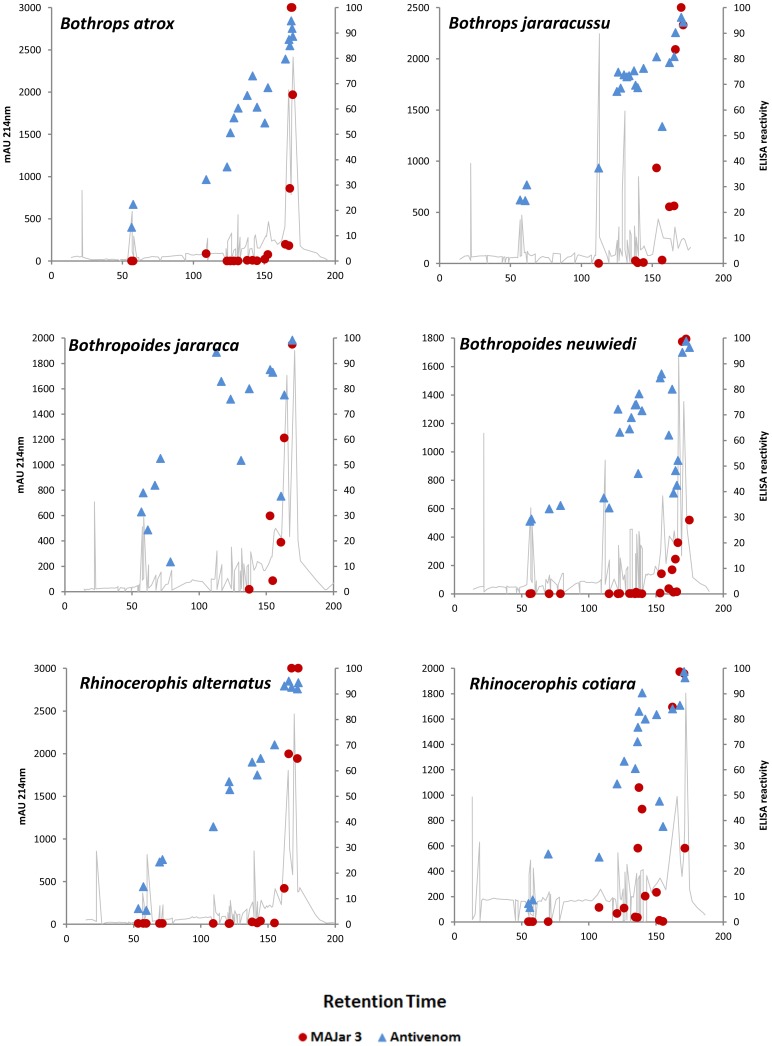
ELISA reactivity with *Bothrops* antivenom and MAJar-3 monoclonal antibody of fractions collected from chromatograms of venom from snakes classified in different genera. Samples containing 100 µL 1-µg/mL fractions collected at the elution times represented in the chromatograms were used to coat maxisorb microplates (Nunc), which were incubated with SAB (1∶1,000) or a monoclonal antibody against jararhagin (class P-III SVMP) MAJar-3 (1∶50), followed by incubation with anti-horse IgG (1∶2,000) or anti-mouse IgG (1∶1,000) labeled with peroxidase. The reactions were developed with ortho-phenylenediamine/H_2_O_2_ as the enzyme substrate, and the products were detected at 490 nm. The ELISA reactivity was calculated as % reactivity, taking as 100% the maximal OD value obtained in each of three independent experiments performed in duplicate.

Our next approach was to evaluate the reactivity of the whole venoms and their isolated fractions with antivenoms. Figure 5 shows the titration curves of the antibothropic serum (SAB) in ELISA plates coated with equal amounts of each venom. The SAB antibody titers were the same, regardless of the antigen used, and they corresponded to a dilution of 640,000. The only differences among the venoms were the values obtained for the 10,000 and 20,000 dilutions of SAB against the *B. jararacussu* venom, which were significantly lower than comparing with other venoms. These dilutions reflect the zone at which the antigen concentration is the limiting factor, and differences in antibody binding may reflect the lower amount of reactive antigens in *B. jararacussu* venom, highlighting the antigenic relevance of P-III SVMPs. Indeed, the region correspondent to bands of approximately 50 kDa, which is the approximate molecular mass of P-III SVMPs, were less intense in the *B. jararacussu* venom electrophoresis than others (Figure 6A). SAB preferentially recognized bands of approximately 50 kDa by western blotting (Figure 6B), confirming the higher immunogenicity of SVMPs class P-III. Bands between 20 and 30 kDa, with masses corresponding to SVSPs and P-I SVMPs, were also recognized by SAB (Figure 6B). The SAB reactivity with each fraction from reverse-phase chromatography was also assessed and compared to the reactivity of a monoclonal antibody, MAJar-3, which recognizes the disintegrin domain of P-III SVMPs [51]. In Figure 7, we demonstrate the strong reactivity of the monoclonal antibody with the fractions eluted after 160 minutes (in all chromatograms), confirming that these fractions correspond to P-III SVMPs. The same fractions were the most SAB-reactive antigens in all venoms, regardless of whether these venoms were included in the immunization pool used to prepare the SAB antivenom. Even for the *B. jararacussu* venom, with a low abundance of SVMPs, the fractions eluted after 160 minutes were the most reactive. Intermediate levels of reactivity were detected with the fractions eluted between 120 and 160 minutes, with very limited reactivity for some, particularly the venoms of *B. atrox* and *B. alternatus*, suggesting a lower antigenicity of P-I SVMPs and SVSPs in relation to the SAB antivenom. Interestingly, three small peaks collected from the *R. cotiara* venom at approximately 140 minutes were strongly reactive with SAB and also with MAJar-3, suggesting the presence of P-III SVMPs in this venom, with distinct structural features and elution profiles. Despite the inclusion of *B. jararacussu* and *B. neuwiedi* venoms in the immunization pool, the reactivity of SAB with their fractions (showing PLA_2_ elution characteristics) from 100 to 110 minutes was moderate. The fractions eluted prior to 100 minutes in all of the chromatograms were poorly recognized by SAB. In other publications, fractions that eluted before 100 min under similar chromatographic conditions corresponded to disintegrins [19], [20], vasoactive peptides [19] or DC fragments of SVMPs [20].

Interestingly, despite the different methods used in this study, our results are comparable to those of Núñez et al. [18] and Calvete et al. [16], who showed the complete immunoprecipitation of PIII-SVMPs, to a minor extent of SVSPs and DC-fragments, and limited immunoreactivity towards PLA_2_ molecules and PI-SVMPs by antivenomics of *B. atrox* venom with commercial antivenoms. Using antivenomics of *B. asper* venom and commercial antivenoms, Gutiérrez et al. [9] also showed complete immunodepletion of P-III SVMPs and partial depletion of PLA_2_s, some serine proteinases, and P-I SVMPs. Correa-Neto et al. [26] approached the same issue by immunomics where the western blots of 2D-gel electrophoresed venoms revealed that antiserum against *B. jararacussu* venom showed higher reactivity to SVMPs and weaker reactivity towards SVSPs and PLA2s, and anti-jararaca serum preferentially recognized SVMPs and SVSPs among other antigens. Both of these sera failed to recognize low-molecular weight proteins [26]. Comparing the different methods, antivenomics is the best choice for a detailed study, since identifications of non-depleted proteins will show exactly the antigens that are partially immunodepleted or non-reactive with the antivenom. However, the method used here has the advantage to allow simultaneous tests of different venoms, at exactly the same conditions, and gives comparable results to antivenomics, thus is appropriate for comparative studies.

Important conclusions arise from these results. It becomes clear that P-III SVMPs are the predominant antigens in the venom of snakes from the *Bothrops* complex. Moreover, at least among the *Bothrops*, SVMPs are cross-reactive antigens that are equally recognized in venoms, regardless of their inclusion in the immunization pool. This is a good indication for antivenom efficacy, as P-III SVMPs are also abundant in most of these venoms and are related to the important symptoms of local and systemic envenomings, such as hemorrhage, the activation of coagulation factors, the inhibition of platelet aggregation and the activation of several factors that lead to local symptoms [35]. Interestingly, P-III and P-I SVMPs share similar catalytic domains and catalytic properties [68], which are involved in most of the toxic activities of SVMPs. Therefore, it is very intriguing that P-I SVMPs are less recognized by the antivenoms than are P-III SVMPs and raises some concerns about the neutralization efficacy of those activities related to the catalytic domain of these molecules. This observation suggests different interpretations: the most immunogenic epitopes of SVMPs may be located within the disintegrin-like or cysteine-rich domains; or catalytic domains of P-III SVMPs are more immunogenic than catalytic domains of P-I SVMPs. For instance, high hemorrhagic activity and the inhibition of platelet aggregation are typical for P-III SVMPs and depend upon disintegrin-like/cysteine-rich domains [69], [70], yet P-I SVMPs are able to induce local reactions [71] and activate coagulation factors [72], which are important effects of snake bites.

SVSPs and PLA_2_s are important toxins involved in the coagulopathy and local effects, respectively, of patients bitten by snakes of the *Bothrops* complex. Thus, the limited reactivity of SAB with these fractions should be addressed. Most SVSPs are thrombin-like enzymes involved in the blood coagulation disturbances induced by venom [33], and this symptom is easily controlled in patients after antivenom administration [73], suggesting that the presence of anti-SVSP antibodies in SAB is appropriate to neutralize the activity. However, PLA_2_s are generally myotoxic or pro-inflammatory [34], and these symptoms are not well neutralized by antivenoms. In the case of SVSPs, it appears that the low levels of antibodies present in SAB are sufficient to neutralize the systemic effects of SVSPs after intravenous administration. In contrast, this does not appear to be the case for the neutralization of the local effects of envenomings induced by PLA_2_s or P-I SVMPs. This lack of efficacy could most likely be dependent upon antivenom biodisponibility at the site of the lesion rather than on the potency of an antivenom against the myotoxic or dermonecrotic components of the venom [74] or the antibody titer against the toxins inducing the local effects.

Another important point observed in this study was the limited reactivity of antivenom with disintegrins and the DC fragments of SVMPs, which are recognized as inhibitors of platelet aggregation [69], [75], and its reactivity with vasoactive peptides. Although they are not presently considered major toxins correlated with the symptoms of envenomings, the additive or synergistic role of these small toxins in snake bite disorders cannot be ruled out. These low molecular mass peptides are known to be weakly immunogenic; however, in antivenomics studies, at least DC fragments and disintegrins were depleted from *B. atrox* [18] and *B. asper* [9] venoms by commercial antivenoms. Nevertheless, the presence of antibodies against such classes of low molecular size toxins in antivenoms should be regarded with more attention.

**Figure 8:**
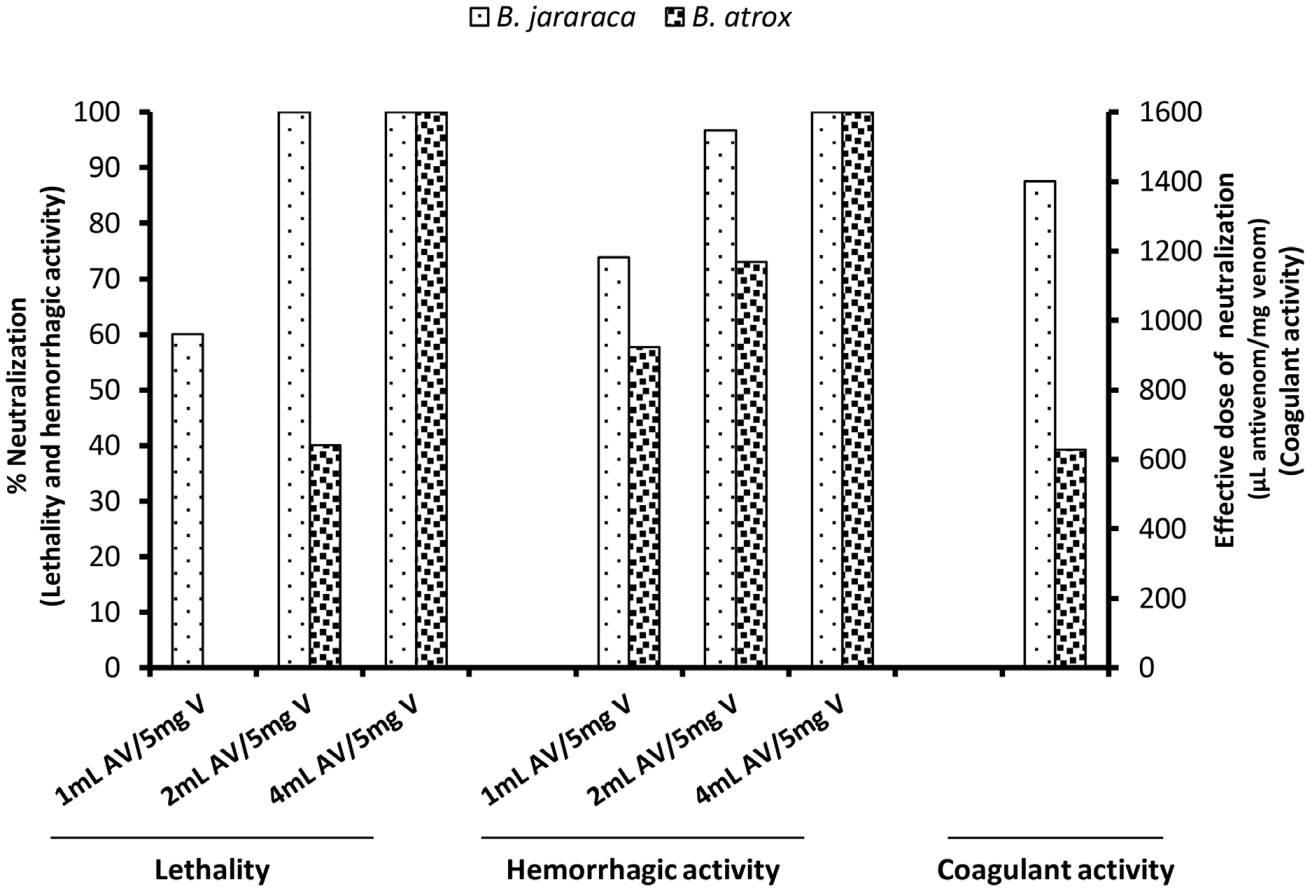
Neutralizing ability of *Bothrops* antivenom (SAB) against the major toxic activities of *Bothropoides jararaca* and *Bothrops atrox* venoms. In the neutralization assays, the *Bothrops atrox* venom was pooled from 8 adult snakes collected in FLONA Tapajós, Santarém, Pará, Brazil. For the neutralization of lethality and hemorrhagic activity, doses of *B. jararaca* or *B. atrox* venoms were pre-incubated with SAB at ratios of 1, 2 or 4 times the SAB volume required to neutralize an equal amount of reference venom according to the manufacturer. To assess hemorrhage, 10 µg was incubated and injected intradermically in the dorsum of a group of 5 mice. The results show the % neutralization of the mean values, taken as 100% activity, of the data obtained after injection with venom incubated with saline. For the neutralization of lethal activity, 3 LD_50_ doses of *B. jararaca* (105 µg) or *B. atrox* (225 µg) venom were incubated, and the mixtures were injected intraperitoneally into groups of 5 mice; lethality was recorded over a period of 48 hours. The results represent the values obtained in 3 independent experiments and are expressed as % neutralization, considering the number of live/dead mice after this period. To assess the neutralization of coagulant activity, a constant amount of venom (2 times the minimum coagulant concentrations) was incubated with several dilutions of antivenom; the mixture was added to 100 µl bovine plasma, and the clotting times were recorded using a model ST4 mechanical coagulometer (Diagnostica Stago). The neutralization was expressed as the effective dose (ED), defined as the antivenom/venom ratio at which the clotting time was increased threefold when compared to the clotting time of plasma incubated with venom alone.

The next step of this study was to evaluate the SAB neutralization efficacy of the lethality, hemorrhagic and coagulant activities of *B. atrox* venom in comparison to *B. jararaca* venom. For these experiments, we used venoms from snakes collected in a region where many accidents are reported. The accepted potency of SAB efficacy, calculated as the volume necessary to neutralize the lethality of standard *B. jararaca* venom, is 1 mL antivenom/5 mg venom. This value was used as a reference to design the neutralization protocols used in this study, whereby this proportion was sufficient to protect more than 50% of mice from the challenge with 3 LD_50_ doses of *B. jararaca* venom (105 µg). However, neutralization of the 3 LD_50_ doses of *B. atrox* venom (225 µg) was achieved only when the proportion of 2 mL antivenom/5 mg venom was used (Figure 8). Most of the standard protocols to assess antivenom potency use a fixed LD_50_ value to challenge experimental mice. Therefore, this is also the reference assay used to compare the antivenom efficacy against different venoms. However, it is important to consider that LD_50_ values are variable among venoms and reflect the toxic activity of each toxin and their synergistic effect to induce death. Additionally, in most tests, the mice are challenged with pre-incubated mixtures of venoms and antivenoms, and, in these reactions, toxins are neutralized or cleared from the solution on a molar concentration basis rather than according to the neutralization of activity. This fact may explain why several previous studies reported that some venoms with higher LD_50_ values, such as *B. atrox* and *B. jararacussu*, are neutralized with higher concentrations of commercial antivenoms.

Similar findings were observed in our study regarding the neutralization of the coagulant activity of *B. atrox* and *B. jararaca* venoms. In this case, the *B. atrox* venom was more coagulant (minimal coagulant concentration in plasma: 10.8 µg/mL) than the *B. jararaca* venom (minimal coagulant concentration in plasma: 35.6 µg/mL), and higher concentrations of *B. jararaca* venom were used in the assays. The SAB neutralized the coagulating activity of both venoms; in this case, however, lower amounts of antivenoms were needed to neutralize the *B. atrox* activity (ED = 627 µL antivenom/mg venom), whereas *B. jararaca* venom neutralization required a higher antivenom concentration (ED = 1400 µL antivenom/mg venom), as shown in Figure 8.

The hemorrhagic activity was comparable between the venoms, and the ratio of 1 mL antivenom/5 mg venom neutralized more than 50% of the hemorrhage induced by both venoms (Figure 8). Taken together, these data suggest that SAB is efficient in neutralizing the most important effects of *B. atrox* venom despite the phylogenetic distance of the snake and the fact that the venom is not included in the immunization pool used to produce SAB. There are previously published papers in the literature suggesting the need to include *B. atrox* venom for horse immunization [76]–[78]. However, our data showing the opposite are supported by a previous study in which SAB immunodepleted the venom proteins from *B. atrox* populations exhibiting the paedomorphic venom phenotype, the same pattern found in specimens collected in Pará State, Brazil [16]. Moreover, our present data are supported by other pre-clinical assessments showing neutralization of the toxic activities of venoms not included in immunization protocols [79], [80] and by a clinical trial for the treatment of snake bite patients clinically classified as mild and moderate in Pará State (Brazil) demonstrated that the efficacy of a conventional antivenom (SAB) was comparable to the efficacy of an experimental antivenom prepared through horse immunization with *B. atrox* venom [81].

Recently, the understanding of venom composition by venomics [5], [6] and tests of the efficacy of antivenoms by antivenomics [8], [9], [11] have been extremely important approaches in order to achieve efficient antivenoms [82]–[84]. In this work, we approached this issue by a multifaceted comparative study of venoms from six species of snakes of distinct phylogenetic clades of *Bothrops* complex. Important differences were observed in venom composition of the snakes from *Bothrops* complex, mainly for *B. jararacussu* venom. However, these differences showed no apparent relationship with the phylogeny of the snakes. In this regard, although the taxonomy of this group is still under revision, the toxins present in the venoms are similar, in agreement with previous molecular data showing that the ancestral genes encoding *Bothrops* major toxin families were already present before the differentiation of the *Bothrops* species [85], [86]. As a result, the antivenom reacted similarly with toxins from the same protein family, as SVMPs, SVSPs or PLA_2_s, regardless of the snake phylogeny or the presence of the venom in the immunization pool used for antivenom production. Thus, we confirm previous data of antivenomics and suggest that it is possible to obtain pan-specific and efficient antivenoms to *Bothrops* snakes through immunization with venoms from a few species of snakes, if immunogenicity and antigenicity of the distinct protein classes of toxins are considered.
